# Frequency of Micturition in Women

**Published:** 1907-07-20

**Authors:** 


### GYNÆCOLOGY.

### FREQUENCY OF MICTURITION IN WOMEN.

In women of middle age frequency of micturition is a common symptom, and it is always of interest to investigate its causation. The term "irritable bladder" which is so often applied to these cases is merely a cloak for ignorance, and has no basis whatever in clinical pathology. Every bladder is eminently irritable, or it would not react to ordinary stimuli. The fact that the bladder expels its contents more frequently than normal for any particular individual, merely means that there is something abnormal stimulating its muscle to act. We do not propose to consider in an exhaustive manner every condition which will cause frequency of micturition, but rather to study those cases which come most frequently before us, and to formulate some rational method of treatment for them. No doubt many women acquire in infant life and puberty the habit of emptying the bladder frequently, but it will be found that this habit applies only to the day-time as a rule; such people have not nocturnal frequency. For this reason one of the most important queries in cases of frequency must be, Is it nocturnal? We shall see, however, that in some instances of frequency that may be classed as pathological, nocturnal frequency does not occur; for instance, in some cases of prolapse of the uterus. Nevertheless, this does not affect the general statement that pathological frequency is nocturnal as well as diurnal. The best way to classify the cases is to consider them under three headings, thus:—

1. Those cases which depend on some change in the urine,
2. Those which depend on some condition outside the bladder, and
3. Those which depend upon actual disease of the urinary tract from the kidney downwards.

By far the commonest cases are those which depend upon some change in the urine. All that is necessary is to make a complete examination of the urine, chemically and microscopically, and in the majority of cases the pathology of the case will be clear. Of course it will not always be easy to see what is the original cause of the urinary change, but, knowing what the change is, the treatment is not very difficult to formulate. Changes in the urine, due to faulty metabolism, are very common, namely, excess of urates, excess of phosphates, and hyperacidity. The changes in the urine which depend on actual diseases of the urinary tract are by no means common, with the single exception of those due to cystitis. Although we cannot say definitely in many instances why a patient has hyperacid urine, or excess of urates or phosphates, there can be no doubt that diet, digestion, and sedentary occupations are often responsible. It is assumed that we are discussing otherwise healthy individuals, and that no febrile disease is present. In these patients a carefully regulated diet with limited meat consumption, attention to the digestive functions and exercise will generally do much towards cure. Medicinally alkalis and hyoscyamus are indicated, the commonly-used formula, potassium citrate, thirty grains, tincture of hyoscyamus, twenty minims, in an ounce of chloroform-water three times a day, being generally efficacious after a week or two. This medicine often succeeds in relieving the backache which often accompanies the urinary symptoms. Sometimes, although the urine shows the above-mentioned changes, this treatment fails to relieve, and it must not be forgotten that there may be some lesion present belonging to the second group in addition to the change in the urine. To investigate the second group a careful abdominal and bi-manual examination must be made. The common lesions outside the bladder which cause frequency are the pressure of tumours on the fundus of the bladder, early pregnancy (though we believe that here changes in the urine cause frequency more often than pressure of the pregnant uterus), prolapse of the uterus (apart from cystitis), unilateral pelvic cellulitis, . and adhesions about the bladder from a past pelvic peritonitis. It is clear that the pressure of a tumour must be removed, prolapse must be treated, and in cases of old peritonitis the bladder must be gradually dilated by daily injections so as to render it more tolerant of distension. The latter cases are most troublesome to treat and require much patience and care. Thus far, the investigation of these cases involves no special knowledge, nor the use of special instruments.

The third group of cases, however, may require the cystoscope for their diagnosis. The only common disease in this group, however, namely cystitis, can be discovered by means of urinary examination. It must not be forgotten that cystitis may occur with acid urine, and that it is only in serious cases of bladder infection that the urine becomes ammoniacal and contains much pus. In many cases the urine is only slightly cloudy, and the presence of small quantities of mucus can only be demonstrated after boiling with caustic potash. In such cases the amount of pus is small and cannot be recognised except by microscopic examination of the deposit after standing in a tall glass, or, better, after centrifugalisation. There should be no difficulty in this, and anyone who possesses a microscope ought to be able to recognise leucocytes in the urine. It is quite uncommon to have recourse to washing out the bladder in mild cases of cystitis in women, the administration of urotropin internally being usually sufficient to effect a cure. It is not always possible to trace the cause of some cases of cystitis, but let it be remembered that vaginal discharges may cause infection of the bladder, apart from prolapse of the uterus or the use of dirty catheters. Prolapse certainly predisposes to cystitis if there is any difficulty in completely emptying the bladder. Calculus, tuberculous ulceration, malignant growths, papillomata, varices at the neck of the bladder, are all comparatively rare conditions in women, and require the cystoscope for their differential diagnosis. Stone in the kidney, renal growths, Bright's disease, diabetes, movable kidney, pyelitis, pyonephrosis and tubercle of the kidney may all cause frequency of micturition, and may all be diagnosed by appropriate examinations of the kidney and the urine.

